# Young Children Respond to Moral Dilemmas Like Their Mothers

**DOI:** 10.3389/fpsyg.2019.02683

**Published:** 2019-12-06

**Authors:** Niklas Dworazik, Joscha Kärtner, Leon Lange, Moritz Köster

**Affiliations:** ^1^Developmental Psychology, Department of Psychology, University of Münster, Münster, Germany; ^2^Differential Psychology and Personality Research, Department of Psychology, Osnabrück University, Osnabrück, Germany; ^3^Cross-Cultural Developmental Psychology, Department of Psychology, Free University Berlin, Berlin, Germany

**Keywords:** moral cognition, trolley dilemma, moral development, universal moral grammar theory, parental influence

## Abstract

There is a large scientific interest in human moral judgments. However, little is known about the developmental origins and the specific role of the primary caregivers in the early development of inter-individual differences in human morality. Here, we assess the moral intuitions of 3- to 6-year-old children and their mothers (*N* = 56), using child-friendly versions of five trolley dilemmas and two control scenarios. We found that children responded to moral dilemmas similar to their mothers, revealed by correlations between the responses of mothers and their children in all five moral dilemmas and a highly similar overall response pattern between mother and child across all judgments. This was revealed by a high agreement in the response pattern of children and their mothers. Furthermore, children’s overall response tendencies were similar to the response tendencies of adults. Thus, similar moral principles (e.g., the Doctrine of the Double Effect) which have been identified in adults, and describes as a universal moral grammar, may guide the moral intuitions in early childhood already. Taken together, the present findings provide the first evidence that children’s moral intuitions are closely associated with the moral intuitions of their mother.

## Introduction

Philosophical thought experiments on moral intuitions have recently been of great scientific and public interest (Mikhail, 2007; Miller, 2008; Bonnefon et al., 2016; Awad et al., 2018), and are taken as indicators for human basic moral principles. While Trolley scenarios, the most widely applied moral dilemma, have been intensely investigated in millions of adults (Mikhail, 2007, 2009; Awad et al., 2018), very little is known about how children judge these dilemmas and how inter-individual differences in moral intuitions develop in early childhood.

In the standard version of the trolley problem, participants are asked to judge the following two scenarios: First, in the Footbridge dilemma, a person is standing on a footbridge and observes a group of five people walking along the train tracks below. When the observer notices that the group is about to be hit by a runaway train, the only way to save the five people would be that the observer shoves a heavy person standing beside the observer off the bridge. This would stop the train and kill the heavy person but save the lives of the five people on the tracks (Thomson, 1985). Usually a minority of participants, about 10–15%, judge this action of the observer as morally acceptable (Greene et al., 2001; Mikhail, 2007, 2009). By contrast, in the Bystander version of the trolley dilemma, the observer can throw a switch and redirect the train onto another track, with only one person walking on it, but that person would then be killed by the train (Foot, 1967). While the outcome of both scenarios remains the same (saving five at the cost of one), the large majority of adult participants, about 90%, agree that in the Bystander dilemma, the switch should be pulled to redirect the train (Mikhail, 2007, 2009).

The different response tendencies across the dilemmas are commonly explained by the interplay of three deontic principles that indicate an underlying universal moral grammar (UMG) (Cushman et al., 2006): First, the Action Principle claims that harm caused by action is morally worse than harm caused by omission (e.g., shoving the big man from the bridge is morally worse than doing nothing). Second, the Contact Principle states that using physical contact to cause harm to a victim is morally worse than causing similar harm without using physical contact (e.g., shoving the big man from the bridge is morally worse than pulling a switch). Finally, the Doctrine of the Double Effect (DDE) argues that harm intended as means to a goal is morally worse than harm foreseen as the side effect of a goal (e.g., shoving a man from the bridge is an intentional action and cannot be interpreted as a side effect, in contrast to pulling a switch).

The general applicability and consistency of the UMG is underlined by the comprehensive work of John Mikhail and colleagues, who tested over 200,000 participants from more than 120 countries (Hauser et al., 2007; Mikhail, 2007, 2009; Miller, 2008). Based on their results, the UMG framework proposes that all humans possess innate tacit knowledge of a variety of legal rules and principles (including those mentioned above), and those are automatically applied to morally relevant situations (Hauser et al., 2007; Mikhail, 2007). Beyond the three principles mentioned above, human morality is assumed to be constituted by a larger conglomerate of deontic rules.

A great body of research, following the tradition of Jean Piaget and Lawrence Kohlberg, has investigated children’s understanding of norms regarding justice, fairness, and equality and the development of moral reasoning from preschool age on (i.e., the retrospective explanations given for moral judgments) (Kohlberg, 1966; Turiel, 1966, 1983; Kohlberg and Kramer, 1969; Nucci and Turiel, 1978). However, these studies apply real-life scenarios and cannot be directly compared to moral intuitions tested in Trolley scenarios and thus to the principles of a UMG as tested in the adult literature. Therefore, we here refer to moral intuitions (a term often used in the literature testing UMG principles (Hauser et al., 2007; Mikhail, 2007; Pellizzoni et al., 2010)) as spontaneous *ad hoc* reactions about the moral quality of a certain action or the omission of this action in a moral dilemma. This is, different to the focus of Kohlberg or Piaget on the argumentative justification for moral decisions, the focus lies here on the differential moral intuitions in different moral dilemma.

A first study on children’s moral intuitions about Trolley scenarios found that preschool children and adults may judge these dilemmas similarly; namely, they advocate pulling the switch in the Bystander scenario, but not in the Footbridge case (Pellizzoni et al., 2010). Another study extended these findings and found that 10-year-old children respond somewhat more utilitarian (i.e., preferring the greater good) in trolley scenarios in contrast to adolescents and adults (Bucciarelli, 2015). These studies only tested two scenarios (Bystander vs. Footbridge) used by Mikhail et al. (Mikhail, 2009), or focused exclusively on school-aged children (Bucciarelli, 2015). In the present study, we used a more extensive set of the commonly applied Trolley scenarios to assess the early development of preschool children’s spontaneous moral judgment and tested whether preschoolers already judge according to the core principles of the UMG (Mikhail, 2009).

Several theoretical approaches in moral psychology propose that human morality is channeled by social influences (Kohlberg, 1966; Damon and Killen, 1982; Haidt, 2001), with a specific role of the family context (Eisenberg and Valiente, 2002). The UMG advocates propose that innate components are foundational to moral intuitions, but also leave room for social and cultural influences on moral intuitions. However, the UMG advocates do not specify the mechanisms that lead to inter-individual variation in moral preferences. To our knowledge, no study compared the moral intuitions of children and their mothers.

Here, we addressed this issue and for the first time, assessed the impact of the family context on the development of the child’s moral preferences. The family context might be one of the critical factors that explain inter-individual variation in moral intuitions. Furthermore, it is still unclear if influences of caregivers in early moral development go beyond real-life scenarios and generalize to abstract scenarios like the Trolley dilemma. Thus, we tested the moral intuition of children and their mothers, to see whether they are associated with children’s intuitions on Trolley dilemmas.

In the present study, we advance our understanding on early development of children’s UMG. First, we extend former research on preschoolers’ moral intuitions and their use of the deontic principles beyond scenarios formerly applied in children. Second, we test if moral preferences of children are related to the preferences of their mothers across a variety of moral scenarios. Our main hypotheses were that moral judgments may be very similar in children like in adults, and that they are constituted in the proximate family context, and, as a consequence, that children’s moral intuitions are similar to the moral intuitions of their mothers.

## Materials and Methods

### Participants

Participants were 56 mother–child dyads from a German urban middle class context (28 girls, 28 boys, *M*_*age*_ = 5.0 years; *SD_*age*_* = 1.0 years, age range: 3.4–6.6 years), recruited via kindergartens. The experimental procedure was conducted in accordance with the World Medical Association’s Declaration of Helsinki (59th WMA general assembly, Seoul, 2008) and informed written consent was obtained from each participant. According to the regulations on freedom of research in the German Constitution (§5 (3)), and the German University Law (§30), this study did not require a separate vote by a local Institutional Review Board.

Additional dyads did not complete all tasks and were thus excluded from further analysis. This was because children were not attentive (*n* = 2), not willing to cooperate with the experimenter (*n* = 1), had language difficulties (*n* = 1) or because mothers did not return their questionnaires (*n* = 11).

### Materials and Procedure

Each child was tested in a quiet room in the kindergarten; the test took approximately 15 min. Mothers received the dilemma questionnaire 4 to 6 months later. All mothers were tested on the same trolley dilemmas as their children. They received illustrated paper versions of the dilemmas, including a dilemma description, identical to the illustrations and dilemma descriptions presented to the children. All mothers were instructed, and confirmed by signature, to answer the questionnaire by oneself without consulting any other person. Questionnaires were returned to a post box in the kindergarten.

In the warm-up phase, the experimenter encouraged the child to tell them about their day. To introduce the experiment, the experimenter asked children whether they liked to hear some stories in which they could decide how the story should end.

**FIGURE 1:**
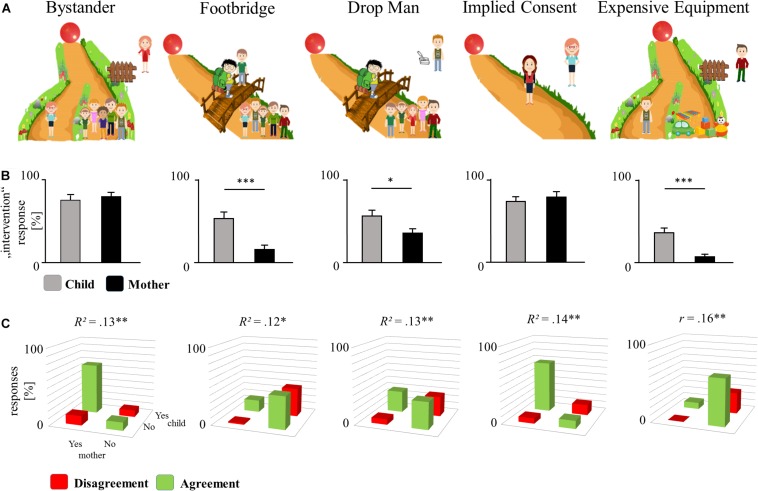
Overview of the trolley dilemma responses. **(A)** The five moral dilemmas shown to mother and child. **(B)** Responses of mothers and children. Bars indicate the percentage of responses that advocate intervention (yes in percent). Whiskers indicate SE. Asterisks indicate the results of McNemar’s tests. **(C)** Correspondence between maternal and children’s advocation for intervention (yes) and for omission (no). Agreements (green) and disagreements (red) between mother–child dyads, with the corresponding correlation coefficient. ^∗^*p* < 0.05, ^∗∗^*p* < 0.01, ^∗∗∗^*p* < 0.001.

**FIGURE 2:**
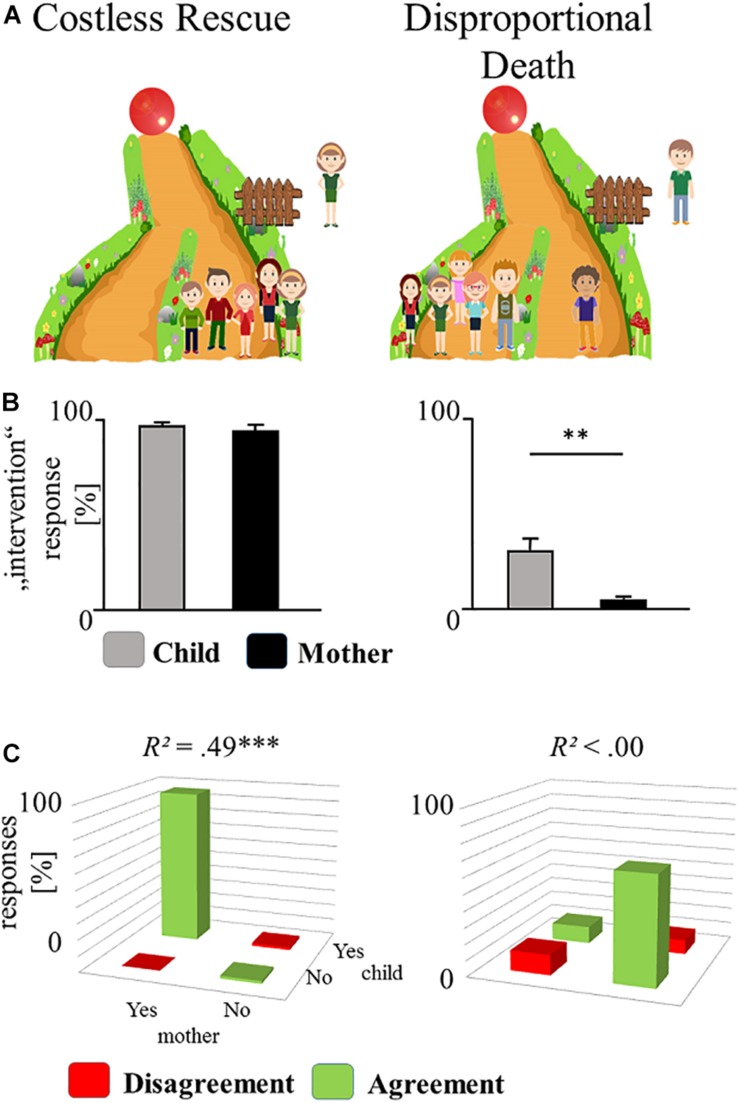
Overview of the control scenarios. **(A)** The two non-moral control scenarios shown to mother and child. **(B)** Responses of mothers and children. Bars represent the percentage of responses that advocate intervention (yes in percent). Whiskers indicate SE. Asterisks indicate the results of McNemar’s tests. **(C)** Correspondence between maternal and children’s advocation for intervention (yes) and for omission (no). Agreements (green) and disagreements (red) of the mother–child dyads with the corresponding correlation coefficient. ^∗∗^*p* < 0.01, ^∗∗∗^*p* < 0.001.

Each child was presented five moral dilemmas and two control scenarios, adapted from Mikhail^3^. The focus was on the five moral dilemmas; the Bystander, Footbridge, Drop Man, Implied Consent, and Expensive Equipment dilemmas (see Figure 1A and description in Table 1). The Disproportional Death and Costless Rescue scenario (see Figure 2A and description in Table 1) were not relevant for our main hypothesis but were assessed as control scenarios, to test whether parents and children were attentive and understood the overall structure of the scenarios. To avoid potential gender biases, the gender of the protagonist was matched to the gender of the child. Furthermore, protagonists were given names different from any child in the kindergarten group.

**TABLE 1:** Dilemma rationale.

	**Scenario description**
**Moral dilemma**	
Bystander	The ball is threatening a group of five children. The agent can redirect the ball onto a side track and thereby save the group from the collision but will hurt one single child.
Footbridge	In contrast to the Bystander case, the only chance to save the five children is to shove a child with a big backpack from the bridge. Thereby that child clashes with the ball and stops it but gets hurt at the same time.
Drop Man	In contrast to the Footbridge scenario, the only way to save the five children is to activate a trapdoor by remote through which the child with the backpack falls onto the tracks.
Implied Consent	In this scenario, a single child is threatened by the ball. The agent can shove the child out of the ball’s way and thereby save the child from getting hurt badly, but the child will still get hurt slightly.
Expensive Equipment	In this scenario, the ball is about to crash into a pile of toys. The only way to save the toys is to redirect the ball onto a side track, where a single child would get hurt by the collision with the ball.
**Control dilemma**	
Costless Rescue	This control condition is similar to the Bystander case. However, no child is standing on the side track, wherefore redirecting the ball does not result in any harm.
Disproportional Death	In this control condition, the ball is threatening a single child. Redirecting the ball onto the side track would result in harming five children.

*See Supplementary Material for the original description of all scenarios.*

For children, all scenarios were presented in a randomized order on a laptop, using PowerPoint (Version 2016, Microsoft). The experimenter explained the plot of the dilemma to the children; the rationale for the seven stories is explained in Table 1 (see Supplementary Material for the full dilemma descriptions). We used control questions up to two times to ensure that children were attentive and understood the basic plot (*Control question 1*: “Can the children see the ball rolling down the hill?”; *Control question 2:* “Is this kid seeing the ball rolling down the hill?”). All children responded appropriately. After the scenario was explained and shown visually to the children, the experimenter explained, for example, in the Bystander scenario: “If the ball will be redirected to the side, this child will get hurt, but the other five children will be saved.” The child was then asked the same test question in all scenarios: “What should Tom/Marie do? Redirect the ball to the side or do nothing?” Therefore, responses were choices for action or for omission. Children (and mothers) responded properly by naming either the action (*Redirect the ball*) which we labeled as a “*Yes*” response in Figure 1B or the omission (*do nothing*), which we labeled as a “*No*” response.

### Statistical Analysis

To test the overall match between the responses of the mother and the child, we conducted an agreement pattern permutation test: The mean agreement between mother and child in the actual sample was tested against the permutation distribution of random mother–child dyads. Specifically, we took the percentage of responses of mothers and their children, which mother and child agreed on (e.g., 60%, if 3 of 5 responses were identical) and compared this to the percentage of responses to 1000 samples of randomly assigned mother–child pairs. That is, we permuted the 56 mother–child allocations 1000 times to estimate the distribution of a chance agreement for random mother–child combinations. A *z* test was then used to test the difference of the agreement in the current sample from the chance distribution (percent of agreements in the random mother–child pairs, as estimated in the permutation test). This way, we compared the agreement in responses of actual mother–child dyads of our sample to a chance agreement, which would occur in randomly assigned mother–child dyads. Note that this chance level estimate effectively controls for the unequal distribution of yes and no responses in each of the dilemma, because it considers the agreements within each dilemma for both the actual and the random mother–child pairs. To determine whether the Doctrine of the Double Effect affected the responses of mothers and children, we compared the Bystander and the Drop Man dilemma by using a McNemar’s test. To test whether the Contact Principle was in effect, we compared the Footbridge and the Drop Man dilemma. Since this was the first study of its sort, testing moral judgments in mothers and their children, we did not conduct an *a priori* power analysis so results of *post hoc* sensitivity power analysis are reported for correlations (Faul et al., 2009). Furthermore, we calculated confidence intervals by converting *r* to Fisher’s *z*′, computing the regarding confidence intervals and converting these back using the inverse Fisher’s *z*′ transform (Beaulieu-Prévost, 2006). Note that, in scenerios where the child or the mother did not respond to a specific scenario (i.e., missing data) those data points were excluded from the analysis.

## Results

### Dilemma Responses

The moral dilemmas and the responses are displayed in Figures 1A,B. We conducted binominal tests against 50% chance level for children and mothers separately for each dilemma. Both mothers and their children advocated to act in the Bystander and the Implied Consent dilemma, all *p*s < 0.001 (binominal test against the 50% chance level). Mothers rather refrained from intervention in the Footbridge, the Drop Man, and the Expensive Equipment dilemmas, all *p*s < 0.058, while the response of children did not show a clear tendency in these dilemmas, all *p*s > 0.081. The dilemma-specific differences in responses between mother and child are displayed in Figure 1B, indicating higher tendencies for intervention in the situation by children in three of five scenarios.

Comparing preschoolers’ responses to the Drop Man and the Bystander dilemmas, the McNemar’s test reached significance (McNemar’s test = 4.81, *p* < 0.019), indicating that children’s responses follow the Doctrine of the Double Effect. The comparison between the Footbridge dilemma and the Drop Man scenario did not reach significance (McNemar’s test = 20.26, *p* = 1.000), which indicates that the Contact Principle is not in effect. For mothers, both comparisons reached significance, all *p*s < 0.013, which clearly indicates that the Double Effect and the Contact Principle are in effect when mothers judge moral dilemmas.

### Correlations With Age

Looking at the correlations with age, children’s tendencies to act in the scenarios increased with age in the Footbridge, the Drop Man, and the Implied Consent dilemma, all *r* > 0.292, all *p* < 0.029, but not in the Bystander and the Expensive Equipment dilemmas, both *r* < 0.246, all *p* > 0.067.

### Mother–Child Agreement

The agreement and disagreement pattern of mother–child dyads for each dilemma is displayed in Figure 1C. The correlation between the responses of mothers and their children were significant and highly consistent for all five dilemmas (Bystander: *r* = 0.364, *p* = 0.006, *R*^2^ = 0.132, 1 −β = 0.839, 95% CI [0.119, 0.567]; Footbridge: *r* = 0.336, *p* = 0.012, *R*^2^ = 0.113, 1 −β = 0.768, 95% CI [0.087, 0.545]; Expensive Equipment: *r* = 0.404, *p* = 0.002, *R*^2^ = 0.163, 1 −β = 0.920, 95% CI [0.167, 0.597]; Implied Consent: *r* = 0.377, *p* = 0.004, *R*^2^ = 0.142, 1 −β = 0.867, 95% CI [0.134, 0.577]; Drop Man: *r* = 0.360, *p* = 0.007, *R*^2^ = 0.130, 1 −β = 0.830, 95% CI [0.115, 0.564]). The overall agreement in the response pattern between mother and child was 71.1%, and was markedly above chance agreement (57.5%), *z* = 5.56, *p* < 0.001 (corresponding to 5.56 *SDs* above the mean of the permutation distribution). Note that this is the result of a non-parametric test against a chance agreement pattern (of random mother–child dyads) and therefore controls for the unequal distributions of yes and no responses in each scenario (see Section “Statistical Analysis” for details). The agreement between mother and child did not increase or decrease with child’s age *r* = 0.051, all *p* = 0.709.

### Control Scenarios

In the control scenarios, mothers and children responded as expected in both dilemmas, opting for the rescue (see Figures 2B,C), all *p*s < 0.011. The responses of mothers and children were not correlated for Costless Rescue *r* = 0.018, *p* = 0.899, *R*^2^ = 0.000, 1 −β = 0.052, 95% CI [-0.237, 0.271] and significantly correlated for the Disproportional Death condition *r* = 0.701, *p* < 0.001, *R*^2^ = 0.491, 1 −β = 0.999, 95% CI [0.530, 0.818]. Note that due to the clear tendencies in responses, this correlation is driven by two matches of “yes” responses and thus not further interpreted.

## Discussion

The present results provide first evidence that children respond to moral dilemmas very similar like their mothers. This is indicated by the consistent agreement between the moral judgments of mothers and children in all five moral dilemmas and the similarity of the overall response pattern between children and adults, as revealed by the response pattern permutation test. Furthermore, this is the first study that applied a broad set of trolley scenarios of the original studies at this early age (Mikhail, 2007). The findings confirm our main hypotheses that children respond to moral dilemmas like their mothers and our data suggest that preschoolers make use of similar deontic principles like adults. Furthermore, similar to a former study (Bucciarelli, 2015), children showed a general preference for the greater good option.

Maternal moral preferences were highly consistent with the response pattern in the existing literature (Mikhail, 2007, 2009), underlining that our child versions captured the deontic status of the original scenarios. Overall, children responded very similar to adult participants in this study and the former studies with adult participants. However, different to a former study on children’s moral judgments (Pellizzoni et al., 2010), preschool children generated somewhat more utilitarian judgments in the Footbridge dilemma. Additionally, our data suggest that preschoolers make use of the Doctrine of the Double Effect but, different to adults, they do not judge according to the Contact Principle. This is indicated by more utilitarian responses in the Bystander compared to the Drop Man dilemma, but no significant difference between the Footbridge and the Drop Man scenario, where the only difference lies in the physical contact used to shove the kid from the bridge. Different to adults, this suggests that the Contact Principle is not yet in effect in preschoolers. Furthermore, children rather advocated for intervention in the Implied Consent dilemma to prevent the kid from greater harm. Hence, preschoolers, just as adults, differentiate between gradual harm and consider it morally more permissible to actively hurt a person in order to prevent that person from greater harm than to omit this act. It is noteworthy that the Implied Consent dilemma is one of two dilemmas where moral preferences between mothers and children are nearly identical and where comparisons do not reach significance. This is also true for the Bystander Dilemma where both mothers and children show a clear tendency for the utilitarian option. Furthermore, a clear majority of the children rated the invulnerability of a threatened protagonist as more important than saving desirable toys. This suggests that preschoolers already accurately distinguish between the invulnerability of much sought-after objects and the invulnerability of persons. Still, more than one third of all children advocated to save the toys in the Expensive Equipment Dilemma, while only two mothers did so. This indicates that more than one third of the children take into account children rather getting slightly injured than desirable toys getting destroyed. In contrast, almost all mothers advocated that children remain physically unharmed and that the toys get destroyed.

Children’s explanations made clear that they understood the stories and anticipated the consequences of each decision. The majority of these explanations focus on “saving the one person’s life” or alternatively “saving the toys”. We thus consider it unlikely that these responses are based on methodological artifacts. Interestingly, despite the more utilitarian responses in children compared to mothers in some of the scenarios, developmental trajectories pointed to even more utilitarian intuitions, indicated by an increase in utilitarian tendencies with age, for the Footbridge, the Drop Man, and the Implied Consent dilemma. Consequently, the agreement within these scenarios would decrease with age since mothers show clear non-utilitarian preferences in these dilemma. The control scenarios indicate that children understood the threat of the ball and the consequences of their action, opting for a costless rescue. In the Disproportional Death scenario, a clear majority advocated not to act, which is in the expected direction, but somewhat less clear than expected. Yet, both the children’s explanations and the control questions give clear evidence that all children understood the scenario.

Overall, our data show a strong correspondence of maternal judgments to those of their children. This was indicated by 13.6% of the variance in children’s behavior explained by the judgment of the mother and the agreement pattern permutation test, which revealed that the specific mother–child dyad agreed to a much higher degree (71.1%) than randomly assigned mother–child dyads (57.5%). While this provides first clear evidence that children judge moral dilemmas like their mothers, it raises the intriguing question how moral intuitions are transmitted between mother and child. One possibility is that the way mothers structure morally relevant situations to their children shapes children moral intuitions from early in development, similar to findings about early concepts of helping behavior (Köster et al., 2015, 2016). However, one could speculate that another viable option might be genetic factors, as indicated by a study that identified genetic variants related to participant’s moral judgments (Walter et al., 2012). The influences identified here are rather strong, given that we only considered the mother (i.e., the moral judgment of the father and close others may also have an influence) and given that the trolley scenarios assess rather abstract moral concepts, which are not close to daily life scenarios. Thus it may also be the case that both socialization and genetic factors play a critical role for the development of moral intuitions, informing the developmental system underlying children’s early socio-cognitive development (Köster and Kärtner, 2019). This is also noteworthy from a theoretical point of view since the UMG advocates notice that there is, at least to some degree, socially transmitted moral diversity; although, they are mute on the mechanisms that enable the development of moral diversity (Mikhail, 2012), p168ff]. In our perspective, the mother–child agreement found in the present study may provide a link between the UMG and the claim of Haidt’s social intuitionist model of moral judgment, by showing that inter-individual variation in moral judgments of children is to some degree transmitted within the family.

For future research, it would be valuable to investigate how moral values may be socialized, for example, by looking at mother–child interactions when discussing moral dilemma together (for a recent study into this direction, see Mammen et al., 2019). It would also be valuable to extend the focus of social influences beyond the family context and to combine socialization studies with genetic analysis to get a better idea about the mechanisms of intergenerational transmission of moral judgments. Moreover, further research should elucidate, how individual’s moral grammar develops over the lifespan (e.g., at what age the Contact Principle develops), throughout the school years, and which additional factors influence moral intuitions throughout development.

To conclude, the findings support our main hypotheses that children respond similar to moral dilemmas like their mothers and make use of similar deontic principles. In this way, the present study opens up interesting avenues for future research, how moral preferences are socialized within the parent–child interaction.

## Data Availability Statement

All data underlying the analyses are provided in the Supplementary Material.

## Ethics Statement

Ethical review and approval was not required for the study on human participants in accordance with the local legislation and institutional requirements. Written informed consent to participate in this study was provided by the participants’ legal guardian/next of kin.

## Author Contributions

ND and MK conceptualized the study. ND, LL, and MK developed the study design and stimuli. LL collected the data. ND, JK, and MK analyzed the data and wrote the manuscript. MK supervised the study. All authors approved the final version of the manuscript for submission.

## Conflict of Interest

The authors declare that the research was conducted in the absence of any commercial or financial relationships that could be construed as a potential conflict of interest.

## Supplementary Material

DATA SHEET S1 Data underlying the final analyses.
